# Inhibition of AKT promotes FOXO3a-dependent apoptosis in prostate cancer

**DOI:** 10.1038/cddis.2015.403

**Published:** 2016-02-25

**Authors:** T P Das, S Suman, H Alatassi, M K Ankem, C Damodaran

**Affiliations:** 1 Department of Urology, University of Louisville, Louisville, KY 40202, USA; 2 Department of Pathology, University of Louisville, Louisville, KY 40202, USA

## Abstract

Growth factor-induced activation of protein kinase-B (PKB), also known as AKT, induces pro-survival signaling and inhibits activation of pro-apoptotic signaling molecules including the Forkhead box O-3a (FOXO3a) transcription factor and caspase in transformed prostate cells *in vitro*. Earlier we reported that Withaferin-A (WA), a small herbal molecule, induces pro-apoptotic response-4 (Par-4) mediated apoptosis in castration-resistant prostate cancer (CRPC) cells. In the present study, we demonstrate that inhibition of AKT facilitates nuclear shuttling of FOXO3a where it regulates Par-4 transcription in CRPC cells. FOXO3a is upstream of Par-4 signaling, which is required for induction of apoptosis in CRPC cells. Promoter bashing studies and Ch-IP analysis confirm a direct interaction of FOXO3a and Par-4; a sequential deletion of FOXO3a-binding sites in the Par-4 promoter fails to induce Par-4 activation. To confirm these observations, we either overexpressed AKT or silenced FOXO3a activation in CRPC cells. Both methods inhibit Par-4 function and apoptosis is significantly compromised. In xenograft tumors derived from AKT-overexpressed CRPC cells, FOXO3a and Par-4 expression is downregulated, leading to aggressive tumor growth. Oral administration of WA to mice with xenograft tumors restores FOXO3a-mediated Par-4 functions and results in inhibited tumor growth. Finally, an inverse correlation of nuclear localization of AKT expression corresponds to cytoplasmic Par-4 localization in human prostate tissue array. Our studies suggest that Par-4 is one of the key transcriptional targets of FOXO3a, and Par-4 activation is required for induction of apoptosis in CRPC cells. Activation of FOXO3a appears to be an attractive target for the treatment of CRPC and molecules such as WA can be explored further for the treatment of CRPC.

AKT is a serine/threonine protein kinase that regulates a major pro-survival signaling network and affects a variety of cellular processes including proliferation, survival, and protein translation,^1^ which makes AKT a potential therapeutic or preventative target for cancer.^2^ Overexpression of AKT has been correlated with the development and metastasis of various cancers.^3, 4, 5^ Activation of AKT disturbs the balance of cell survival and apoptosis by promoting pro-survival transcription factors (CREB and NF-KB) and inhibiting the FOXO3a pro-apoptotic transcription factor.^6, 7, 8^

FOXO3a has a pivotal role in both oncogenesis and tumor suppression.^9^ Loss of FOXO3a has been observed in various cancers, and its cellular localization and phosphorylation status are considered to be prognostic factors for breast,^10^ prostate,^11, 12^ bladder,^13^ and ovarian cancer.^14^ Activated FOXO3a triggers cell-cycle arrest and apoptosis through expression of genes necessary for cell death,^15^ including Fas ligand (FasL), tumor necrosis factor-related apoptosis-inducing ligand (TRAIL), and *Bim*.^16^ Overexpression of FOXO proteins induces apoptosis in cells of various tissue types and possesses tumor-suppressor functions.^17^ Together, these studies suggest that FOXO3a functions as a tumor suppressor, and therefore may serve as either a direct or indirect target for cancer therapy.^18^

Prostate apoptosis response-4 (Par-4), also known as PAWR, is a tumor-suppressor protein that is reported to cause apoptosis in cancer cells^19, 20^ by activating both intrinsic and extrinsic pathways.^21^ Par-4 is expressed in diverse normal and cancerous cell types and tissues, and resides in both the cytoplasm and the nucleus. Downregulation of Par-4 is considered to be critical for tumorigenesis as it is downregulated in various human cancers including prostate cancer (CaP).^22^ Par-4 knockout mice spontaneously develop tumors in various organs^21^ and exhibit prostatic intraepithelial neoplasia.^23^ In order for endogenous levels of Par-4 in normal and cancer cells to cause apoptosis, an additional stimulus is required.^24, 25, 26^

CaP is the third leading cause of cancer-related death in men in the United States.^27^ Most patients initially respond to hormone ablation therapy. However, some patients eventually become refractory to such treatments, having developed what is known as castration-resistant prostate cancer (CRPC).^28^ Par-4 modulation has tremendous therapeutic potential and, indeed, genetic or pharmacological strategies to induce Par-4 expression are currently under investigation for cancer prevention or treatment.^29^ Withaferin-A (WA) is a natural bioactive compound derived from the roots and stems of *Withania somnifera,* which is cytotoxic in several cancer cell lines.^30, 31^ Our previous reports confirmed that Par-4 is a critical downstream target for WA treatment to execute its anticancer effect against CRPC.^32^ Dissecting signaling cascades upstream of Par-4 might allow us to identify potential therapeutic targets that can restore Par-4 function in CRPC cells.

In the present study, we demonstrate that Par-4 is one of the downstream transcriptional targets of FOXO3a that executes pro-apoptotic function in CRPC cells. FOXO3a directly interacts with the Par-4 promoter and activates and initiates caspase-mediated cell death upon WA treatment. Small molecules such as WA can restore the FOXO3a and Par-4 signaling axis and apoptosis, which could be an attractive target for treatment of CRPC.

## Results

### WA inhibits AKT signaling and induces Par-4 activation in AR-null CRPC cells

Inhibition of pAKT expression was evident in WA-treated cells (PC-3 and DU-145) and induced Par-4 expression in a time- and dose-dependent manner (data not shown) by western blot analysis (Supplementary Figure S1A). GSK-3*β* acts as a downstream effector of AKT that executes AKT-induced cell growth, proliferation, and survival in many cancer types, including CaP cells; hence, we analyzed GSK-3*β* expression in CaP cells.^33, 34^ A decrease in phosphorylated GSK-3*β* expression was observed in WA-treated CRPC cells (Supplementary Figure S1A) in a dose- (data not shown) and time-dependent manner similar to that of Par-4 induction (Supplementary Figure S1A). Consistently, upregulation of Par-4 transcription (2.5- to 7-fold) and promoter activation (2- to 4-fold) was observed in both CRPC cell types (Supplementary Figure S1B and C). Inhibition of AKT or induction of Par-4 by WA in both CRPC cell lines resulted in WA-induced dose-dependent growth inhibition in both cell lines (Supplementary Figure S1D). Together, these results reveal that WA inhibits AKT activity and induces Par-4, which correlates with WA-induced cytotoxicity in CRPC cells.

### Inhibition of AKT negatively regulates Par-4 function in AR-null CRPC cells

**Figure 1:**
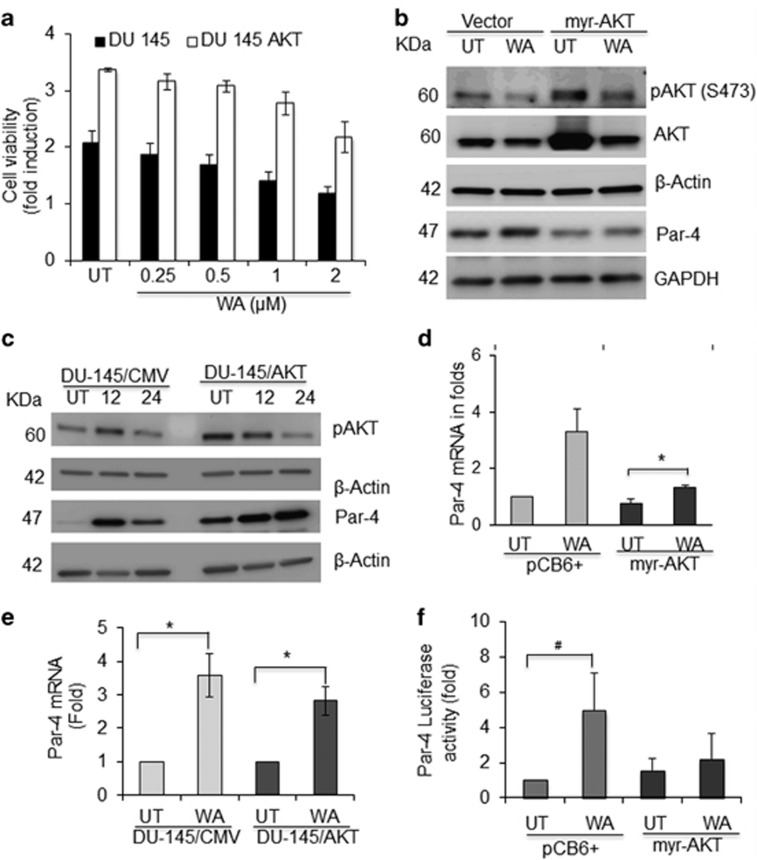
AKT overexpression attenuates the effect of Par-4. (**a**) Effect of WA treatment on cell viability of DU-145, and DU-145/AKT cells for 24 h. The control cells were treated with DMSO or with the indicated concentration of WA for 24 h. Bars represents mean of three experiments with S.E. (**b**) PC-3 cells were transiently transfected with myr-AKT and empty vector. After transfection, cells were treated with or without WA 2 *μ*m. After 24 h, cells were harvested and cell lysates were prepared. Total cellular lysates were subjected to western blot analysis using antibodies against pAKT, AKT, and Par-4 proteins. *β*-Actin was used as a loading control. (**c**) DU-145/CMV and DU-145/AKT cells were treated with or without WA at a concentration of 2 μm concentration. Total cellular lysates were prepared and subjected to western blot analysis using antibodies against pAKT, AKT, and Par-4 proteins. *β*-Actin was used as a loading control. (**d**) RT-PCR showing Par-4 mRNA levels with WA treatment in PC-3 cells transfected with or without myr-AKT. (**e**) DU-145 and DU-145/AKT cells were treated with WA for 12 and 24 h, and RNA was isolated and subjected to RT-PCR analysis. (**f**) PC-3 cells were cotransfected with Par-4 promoter luciferase reporter construct, myr-AKT expression plasmid construct with renilla CMV as transfection control, and/or treated with WA. After 24 h, cells were harvested and assayed for luciferase reporter activity. Significant difference from control values was indicated at *P*<0.05 (Student's *T*-test). *P<0.05 and ^#^*P*=0.06

To elucidate the functional role of Par-4 in response to AKT signaling, we either transiently transfected myr-AKT or stably transfected total AKT into CRPC cells and studied AKT-mediated Par-4 function in response to WA treatment. Cell viability assays suggest that AKT-overexpressed cells grow much faster (~1.2-fold in PC-3 (data not shown) and 1.5-fold in stable AKT/DU-145) than vector-transfected CRPC cells (Figures 1a and b). WA treatments significantly overcome AKT-mediated growth induction in both PC-3 and DU-145 cells (Figures 1a and b).

WA treatment induced Par-4 in vector-transfected cells; however, WA partially rescues Par-4 expression in AKT-overexpressed cells (Figure 1c). However, a higher concentration of WA completely downregulates pAKT expression and upregulates Par-4 function in CRPC cells (data not shown). Similar results were found in stably overexpressed AKT/DU-145 cells (Figure 1e). However, WA treatment restored Par-4 mRNA expression (Figure 1d) and partially promoter activity in PC-3 cells (Figure 1f).

### Molecular link between FOXO3a and Par-4 in AR-null CRPC cells

**Figure 2:**
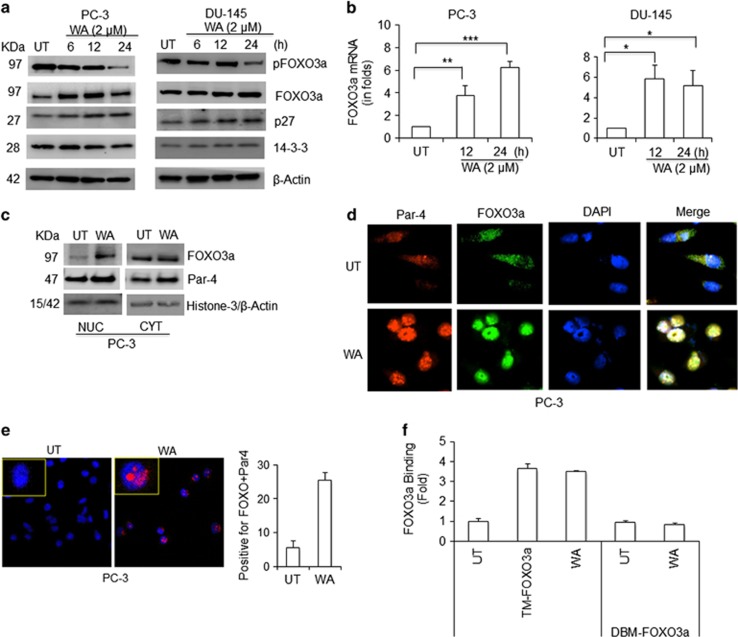
FOXO3a and Par-4 induction and nuclear localization after WA treatment. (**a**) Time-dependent effect of WA treatment on FOXO3a, pFOXO3a (Ser253), p27, and 14-3-3 proteins in PC-3 and DU-145 cell lines. (**b**) WA effect on FOXO3a mRNA expression. (**c**) Cytoplasmic and nuclear extracts isolated from PC-3 cells treated with WA and subjected to western blotting for FOXO3a and Par-4 expression. (**d**) Confocal microscopy showing FOXO3a and Par-4 nuclear localization in control *versus* WA-treated PC-3 cells. (**e**) PC-3 cells were treated with or without WA and immunostained with olink probes for FOXO3a and Par-4 proteins. Nuclei were stained with DAPI. Quantitative analyses were done by counting the number of positive cells showing red dots. (**f**) PC-3 cells were transfected with HA-FOXO3a-TM and HA-FOXO3a-DBM. Lysates were collected and allowed to bind to the coated biotinylated oligos containing FOXO3a-binding sites. Using anti-HA AP conjugated secondary antibodies, bound proteins were quantitated by colorimetric assay

WA inhibited pFOXO3a(ser253) expression and allowed total FOXO3a accumulation in CRPC cells. Endogenous FOXO3a activation levels in these cells were determined by analyzing the expression of p27, which is a known downstream target of FOXO3a. Increased time-dependent expression of p27 suggested that WA induced FOXO3a function in CRPC cells (Figure 2a). No alteration in 14-3-3 expression was seen in WA-treated CRPC cells (Figure 2a), suggesting that FOXO3a accumulation in the nucleus is not due to inhibition of 14-3-3. FOXO3a transcription is upregulated by 4- to 5-fold compared with vehicle-treated control following WA treatment of both PC-3 and DU-145 cells (Figure 2b).

Higher levels of FOXO3a transcription and accumulation of FOXO3a expression were seen in both nuclear and cytoplasmic compartments in WA-treated cells as compared with vehicle-treated PC-3 cells (Figure 2c). A concomitant nuclear accumulation of Par-4 was also seen in WA-treated cells (Figure 2c). In immunofluorescence studies, as expected, WA-treated cells exhibited co-localization of both FOXO3a and Par-4 in the nucleus, signifying that to execute their pro-apoptotic function both proteins should be localized in the nucleus (Figure 2d). In proximity ligation assays, which are used to visualize nuclear co-localization events,^35^ co-staining of WA-treated CRPC cells with FOXO3a and Par-4 antibodies showed an appearance of red dots in the nucleus, which implies the close proximity of the two proteins. In control cells, no distinct red dots appeared (Figure 2e). In DNA binding studies (EMSA), TM-FOXO3a (transcriptionally active) was used as a positive control and DBM-FOXO3a (DNA binding mutant) as a negative control. Maximum binding efficacy was observed in WA-treated cell extracts as well as in TM-FOXO3a-overexpressing cells as compared with control cells, suggesting that nuclear FOXO3a binds directly to Par-4 promoter regions (Figure 2f). These results suggest that WA concomitantly activates both FOXO3a and Par-4 signaling in CRPC cells.

### FOXO3a activation is essential for WA-induced Par-4 function in AR-null CRPC cells

**Figure 3:**
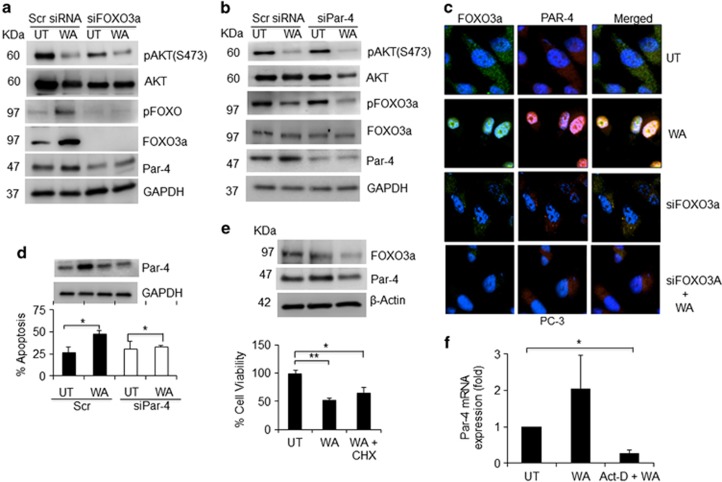
Par-4 expression is inhibited by siRNA against FOXO3a. (**a** and **b**) PC-3 cells were transiently transfected with siFOXO3a, siPar-4, and scrambled siRNA and followed with WA treatment. After 24 h, total cellular lysates were prepared and subjected to western blot analysis for AKT, pAKT (ser473), FOXO3a, pFOXO3a (Ser253), and Par-4 proteins. GAPDH was used as a loading control. (**c**) Confocal microscopy showing the expression of FOXO3a and Par-4 proteins. PC-3 cells were transiently transfected with siFOXO3a, and scramble siRNA with or without WA treatment. Lower, FOXO3a and Par-4 proteins in WA-treated or control cells were immunostained with primary and the corresponding FITC- or TRITC-conjugated secondary antibodies followed by detection using confocal microscopy. Green signals indicate FOXO3a, whereas red signals indicate Par-4. Nuclei were counterstained with DAPI. Representative images of each sample are shown. (**d**) PC-3 cells transfected with siRNA for Par-4 and treated with or without WA for 24 h and stained with annexin-FITC and PI nuclear stain and scored for apoptosis analysis. (**e**) PC-3 cells were treated with or without WA after 8 h pre-incubations with 1 *μ*g/ml final concentrations of cyclohexamide. After 24 h, cells were harvested and analyzed for FOXO3a and Par-4 proteins. Cell viability was measured by MTT assay for WA, and WA+CHX-treated PC-3 cells. (**f**) PC-3 cells were pretreated with transcriptional inhibitor actinomycin-D at 10 *μ*g/ml concentration before WA treatment for 12 h, and total RNA was subjected to RT-PCR analysis. Significant difference from control values was indicated at *P*<0.05 (Student's *T*-test). *P<0.05 and **P<0.005

To determine whether FOXO3a activation is an upstream or downstream event of Par-4 transcription, we silenced either FOXO3a or Par-4 with small interfering RNA (siRNA) in CRPC cells, which were then subjected to WA treatment. Silencing FOXO3a downregulated Par-4 expression in control cells and in WA-treated cells (Figure 3a). By contrast, in Par-4 silenced cells, no alterations in the levels of FOXO3a or pFOXO3a were observed (Figure 3b). Immunofluorescence data suggest that silencing FOXO3a expression before WA treatment inhibited Par-4 expression as well as nuclear localization (Figure 3c). Phenotypic analysis further confirmed that induction of FOXO3a is necessary for Par-4 mediated pro-apoptotic function in CRPC cells (Figure 3d). To ascertain that FOXO3a is required for Par-4 activation, WA-treated CRPC cells were treated with or without the protein synthesis inhibitor cyclohexamide (CHX) or transcriptional inhibitor actinomycin-D. Immunoblotting showed WA-induced FOXO3a and Par-4 expression, and the cells pretreated with CHX failed to induce FOXO3a and Par-4 expression (Figure 3e), suggesting that newly synthesized FOXO3a may be responsible for Par-4 expression. Similarly, Par-4 mRNA was practically abolished in the presence of actinomycin-D, which validates the post-transcriptional blockage of Par-4 expression by WA (Figure 3f).

Transactivation domain-truncated CT-FOXO3a plasmid was transiently transfected followed by WA treatment. Western blot analysis showed downregulation of Par-4 expression in the presence of WA in CT-FOXO3a-overexpressed cells as compared with vector-transfected cells (Supplementary Figure S2A). Furthermore, immunofluorescence data revealed that WA fails to induce Par-4 expression as well as nuclear localization in CT-FOXO3a-transfected cells, suggesting that Par-4 transactivation was compromised by CT-FOXO3a (Supplementary Figure S2B). Inhibition of Par-4 promoter activity was seen in CT-FOXO3a-transfected cells when compared with controls and WA fails to rescue Par-4 activation in CT-FOXO3a cells (Supplementary Figure S2C). CT-FOXO3a-transfected cells showed resistance to WA treatment in cell viability assays, suggesting that FOXO3a transactivation may be required for Par-4-mediated cytotoxicity in CRPC cells (Supplementary Figure S2D). Overall, these results suggest that Par-4 signaling is downstream of FOXO3a signaling, and FOXO3a activation is essential for Par-4 function in CRPC cells.

### Overexpression of FOXO3a mimics WA and induces Par-4-mediated cell death in AR-null CRPC Cells

**Figure 4:**
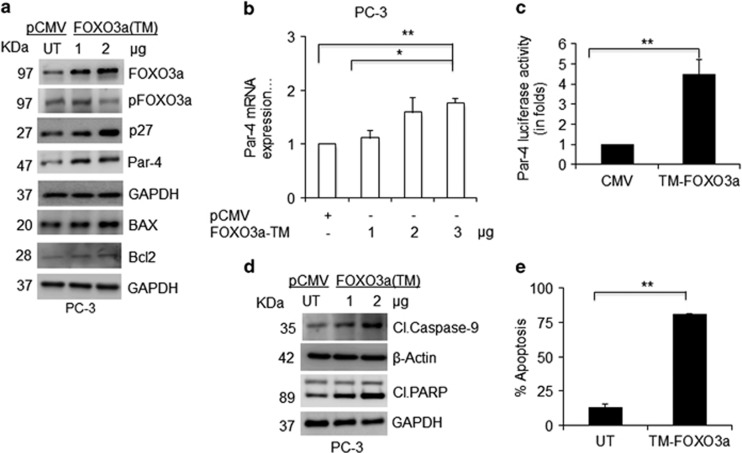
Activation of FOXO3a induces Par-4 mRNA expression and apoptosis. (**a**) PC-3 cells were transiently transfected with FOXO3a (TM) plasmids and an empty vector. After 36 h, cell lysates were prepared, and expression patterns of HA-FOXO3a, pFOXO3a (Ser253), Par-4, BAX, Bcl-2, and p27 were analyzed by western blot analysis. (**b**) RT-PCR showing the fold increment in Par-4 levels with TM-FOXO3a plasmid overexpression. (**c**) Effect of FOXO3a-TM on Par-4 promoter activity. (**d**) Lysates showing the activation of pro-apoptotic proteins Cl-PARP, Caspase-9, and GAPDH/actin was analyzed by western blotting. (**e**) PC-3 cells were transfected with TM-FOXO3a expression plasmid and stained with annexin-FITC and PI nuclear stain and scored for apoptosis analysis. Significant difference from control values was indicated at *P*<0.05 (Student's *T*-test). *P<0.05 and **P<0.005

To confirm the pro-apoptotic role of FOXO3a, we transiently overexpressed TM-FOXO3a in CRPC cells. A dose-dependent expression of FOXO3a protein as well as Par-4 was observed. Overexpression of FOXO3a activated Par-4 downregulates Bcl-2 expression and upregulates BAX expression. Further, upregulation of p27 confirmed activation of FOXO3a in CRPC cells (Figure 4a). Real-time PCR analysis showed that FOXO3a transcriptionally regulates Par-4 gene expression in CRPC cells (Figure 4b). In luciferase reporter assay, transfection of TM-FOXO3a itself showed 4-fold Par-4 transcriptional activity (Figure 4c). Earlier, we reported that Par-4 induces the caspase signaling cascade to execute cell death, so we examined caspase signaling in TM-FOXO3a-overexpressing cells. TM-FOXO3a-transfected cells showed increased apoptosis, which corresponds to caspase-9, and PARP cleavage, suggesting that activation of FOXO3a directs cell death in CRPC cells similarly to WA cell treatment (Figures 4d and e). These results imply that overexpression of FOXO3a mimics the effect of WA in CRPC cells.

### Transcriptional regulation of Par-4 by FOXO3a

**Figure 5:**
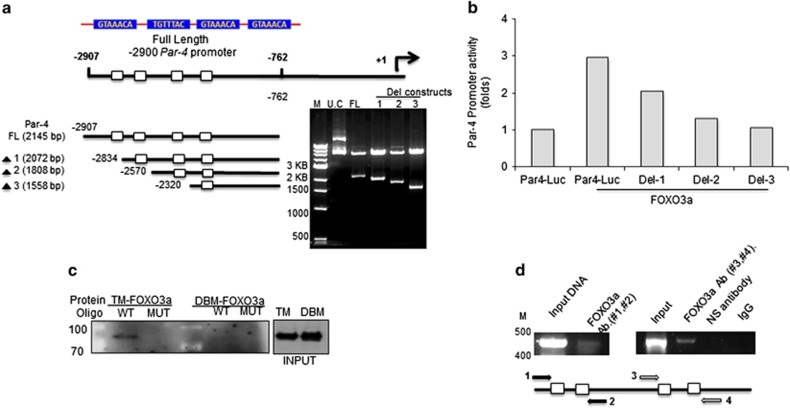
Activation of FOXO3a induces *Par-4* gene promoter activation. (**a**) DNA region showing Par-4 promoter with four consensus FOXO3a DNA binding sites spanning between +1 and −2900 bps. Below are the comparisons of various Par-4 deleted regions constructs with an ability to transactivate the Par-4 promoter region upon FOXO3a transfection or WA treatment. (**b**) PC-3 cells were transfected with 2.1 Kb Par-4 full length and deletion constructs (Del-1; 2.0 KB, Del-2; 1.8 KB and Del-3; 1.5 KB) promoter luciferase reporter constructs with renilla CMV as a transfection control, and/or transfected with FOXO3a expressing plasmid. Then, cell lysates were assayed for Luciferase reporter activity by showing fold induction of Par-4 activation. (**c**) PC-3 cells were transfected with HA-tagged FOXO3a TM or H212R mutant. The cell lysates were incubated with biotinylated DNA probes encoding WT or Mutated Par-4 promoters and pull-down assay was performed. FOXO3a was detected by immunoblotting with anti-HA to detect HA-tagged FOXO3a proteins. (**d**) Ch-IP analysis of FOXO3a binding to the *Par-4* promoter region. PC-3 cells were transfected with FOXO3a and its genomic content was immunoprecipitated with anti-FOXO3a or control IgG. Immunoprecipitates from WT and FOXO3a-transfected cells were analyzed by quantitative PCR, using primers corresponding to Par-4 promoter

Potential FOXO3a binding sites (100% homology) at position -2841; GTAAACA, -2577; TGTTTAC, -2327; GTAAACA and -2106; GTAAACA) with start codon were identified in *Par-4* promoter (GenBank ID: AF503628.1) by bioinformatics analysis. PCR amplified -762 to -2907 (2.1 KB) region of the *Par-4* promoter was used to generate full-length reporter construct. The sequential deletions of FOXO3a-binding sites within -762 to -2907 (2.1 KB) region were used to generate deleted reporter constructs spanning from −762 to – 2834 (2.0 KB); −762 to −2570 (1.8 KB); −762 to −2320 (1.5 KB). To confirm further FOXO3a mediated *Par-4* regulation, we over expressed FOXO3a, which activated significantly *Par-4* promoter, however sequential deletion of FOXO3a binding sites in each deletion constructs showed a gradual loss of Par-4 promoter activity (Figure 5b).

To confirm whether FOXO3a directly binds to *Par-4* promoter, DNA pull-down assay was performed. Cells that expressed TM-FOXO3a showed significant binding to the oligonucleotides containing wild-type FOXO3a-binding sequences corresponding to the Par-4 promoter binding sites, while DBM-FOXO3a failed to bind. These data provide further evidence suggesting that FOXO3a has potential binding sites available in the *Par-4* promoter (Figure 5c). We next performed chromatin immunoprecipitation (Ch-IP) assays to determine the FOXO3a occupancy on the *Par-4* promoter. As demonstrated in Figure 5d, an increased binding of FOXO3a to this *Par-4* promoter region is consistent with the DNA pull-down results. Overall, all three experiments confirm that FOXO3a transcriptionally regulates *Par-4* expression in CRPC cells.

### Xenograft models: Overexpression of AKT induces aggressive tumor growth and WA overrides AKT-mediated growth and restores Par-4 function

**Figure 6:**
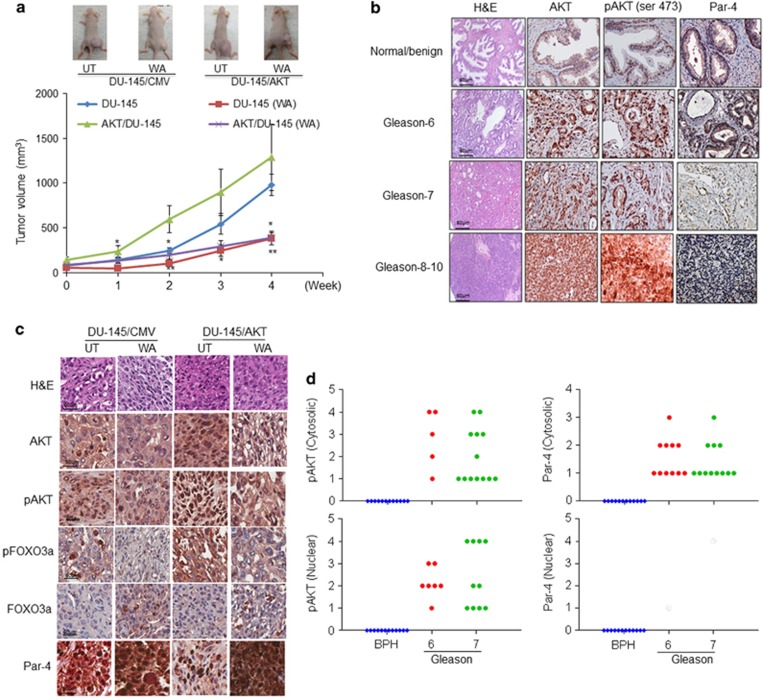
Effect of WA on AKT induced tumors. (**a**) For xenograft studies, 2.5 × 10^6^ DU-145/pCMV or DU-145/AKT cells in a final volume (50 μl) were injected subcutaneously in either the left or right flanks of mice. The mice were monitored twice weekly, and tumor volumes were measured once a week for 4 weeks. A line graph was plotted to compare tumor growth and volume (mm^3^) for DU-145/pCMV and DU-145/AKT tumors. (**b**) TMA human samples with different grades (Gleason score) showing the pAKT, AKT and Par-4 antigens expression. (**c**) As mentioned in the figures, IHC was performed in vehicle and WA treated DU-145/CMV and DU-145/AKT xenografts. (**d**) Dot plot showing the prevalence of nuclear pAKT and cytoplasmic retention of Par-4 in higher Gleason score of CaP patients as in comparison with normal/BPH controls. *P<0.05 and **P<0.005

CRPC cells that stably expressed AKT/DU-145 and/or pCMV/DU-145 were implanted subcutaneously into the right flank of nude mice and tumors were allowed to grow. Tumor growth was monitored once a week. When the tumor volume reached 50 mm^3^, animals were assigned to one of the two groups. One group received sesame oil or WA (4 mg/kg/body weight) by oral gavage for 4 weeks. Intriguingly, AKT-overexpressing tumors showed 2- to 3-fold faster growth than pCMV-expressing CRPC tumors (Figure 6a). Oral administration of WA significantly reduced AKT-induced xenograft tumor growth. H&E staining in AKT-overexpressing tumors exhibited an increase in the mitotic index (3- to 4-fold) as compared with pCMV-transfected tumor samples (Figure 6b). Similarly, we found that WA-treated both tumors (pCMV and AKT transfected) showed a marked induction of necrosis (10–15%) as compared with vehicle-treated tumor tissues.

Immunohistochemistry of tumor sections revealed high expression as well as nuclear localization of pAKT followed by low levels of Par-4 in sections from AKT-overexpressing tumors as compared with pCMV-expressing tumors. Increased and intense nuclear localization of Par-4 and decreased expression of AKT and pAKT were observed in WA-treated AKT-overexpressing tumors, suggesting an AKT-Par-4 axis. By contrast, pCMV tumor sections derived from WA-treated animals showed less expression of activated AKT and more nuclear-localized Par-4 (Figure 6c).

To confirm that activated AKT negatively regulates Par-4 function and to validate our *in vitro* and *in vivo* findings, we performed an immunohistochemical analysis of pAKT and Par-4 expression on human prostate tissue microarrays (TMAs). The TMA contains normal benign or hyperplasia samples (BPH) (*n*=12) and prostate tumor samples of different grades (*n*=32). pAKT expression was evident in 7/16 (43%) grade I (Gleason 6) and II (Gleason 7) tumor samples and in 68.75% of grade III (Gleason 8–10) tumor samples (11/16). On the other hand, in BPH the expression of pAKT was confined to the basal and secretory layers of the cytoplasm. Similarly, nuclear Par-4 expression was detected in the basal layers of BPH, and cytoplasmic accumulation was seen in secretory cells. Interestingly, in a variety of tumor stages, Par-4 was localized in the cytoplasm, indicating that the pro-apoptotic machinery was compromised in tumor samples (Figure 6d).

## Discussion

Oncogenic activation of AKT regulates many signaling cascades that control cell proliferation, survival, growth, inhibition of apoptosis, and resistance to current drug therapies.^36, 37^ Here, we demonstrate that AKT activation inhibits the pro-apoptotic function of Par-4 by suppressing FOXO3a activation in CRPC cells. In addition, the results revealed that Par-4 is one of the downstream targets of FOXO3a and is dependent on FOXO3a to activate its pro-apoptotic functions in CRPC cells.

In our results, we found that WA inhibits AKT activation by inhibiting phosphorylation at Ser^473^, which results in downregulated phosphorylation of its downstream substrate GSK-3*β*, confirming the suppression of AKT kinase activity. AKT phosphorylates Par-4 and sequesters it in the cytosol, thereby preventing its pro-apoptotic function.^38^ In contrast, PKA phosphorylates Par-4 at T163, which activates the pro-apoptotic functions of Par-4 to induce apoptosis.^19^ We delineated the molecular circuit between AKT and Par-4 by either transiently or stably overexpressing AKT and measuring Par-4 expression and function in CRPC cells. AKT-overexpressed CRPC cells proliferated at a higher rate by transcriptionally downregulating Par-4 expression and function. The cell viability and apoptotic assays suggested that treatment with WA partially rescued AKT-mediated resistance to apoptosis in CRPC cells by upregulating Par-4 function. Similar studies have shown that by inhibiting AKT with siRNA, LY294002 or KD-AKT, Par-4 mediated pro-apoptotic function was rescued in CaP cells.^38^

In our studies, inhibition of AKT activation correlates with concomitant induction of FOXO3a and Par-4, so we investigated the molecular link between FOXO3a and Par-4 in CRPC cells. Activated AKT phosphorylates FOXO3a at Thr^32^, Ser^253^, or Ser^315^ residues and inhibits pro-apoptotic activity such as cell-cycle arrest by downregulating p27, GADD45, and BIM expression.^39, 40, 41^ Loss of FOXO3a function may lead to the suppression of apoptosis^42^ and failure of cell-cycle arrest, which may lead to tumorigenesis.^41^

The inverse expression patterns of Par-4 and pAKT are associated with FOXO3a phosphorylation status. To confirm the functional status of FOXO3a in CRPC cells, we measured the expression of p27, a downstream target of FOXO3a in CRPC cells. EMSA studies confirmed nuclear localization of FOXO3a occurs in response to WA treatment. Furthermore, DBM-FOXO3a abolishes WA-induced Par-4 activation in CRPC cells. Although our studies confirmed a concomitant localization of FOXO3a and Par-4 in the nucleus, PLA-based immunofluorescence confirmed that FOXO3a and Par-4 were situated in co-proximity. Activation of FOXO3a also increased caspase activity and promoted apoptosis in endothelial cells^43^ and in leukemia cells.^44^ These results concur with our findings that activation of FOXO3a induces pro-apoptotic function by triggering Par-4 function.

Similar results were achieved with DBM-FOXO3a- or CT-FOXO3a-overexpressed cells that fail to activate *Par-4* reporter expression. The direct binding of FOXO3a to sites on the *Par-4* promoter was further confirmed by sequentially deleting FOXO3a-binding sites, which fails to activate WA-induced Par-4 function in CRPC cells. FOXO3a is known to transactivate various pro-apoptotic genes.^41, 42, 43, 45^ We have for the first time demonstrated that Par-4 is one of the downstream pro-apoptotic effectors in CRPC cells by sequentially deleting FOXO3a-binding sites in the *Par-4* promoter. These results were similar to findings that FOXO3a activates *Bim* and induces apoptosis.^46^

We further confirmed that inhibition of AKT triggers FOXO3a-mediated Par-4-induced cell death in xenograft models. Treatment with WA results in inhibition of AKT and induction of FOXO3a-dependent Par-4-mediated growth inhibition in vector- as well as in AKT-overexpressed tumors. To confirm the inverse relationship of pAKT and Par-4 expression in human prostate specimens, we performed immunohistochemistry analysis on human prostate TMAs. The results clearly suggested that Par-4 is expressed in BPH whereas pAKT expression was mostly localized in the cytoplasm. We also observed that pAKT is gradually overexpressed in grade-specific manner, whereas Par-4 was completely absent in the tumor specimens. More specifically, nuclear pAKT abrogated Par-4 nuclear localization in tumor tissues. We understand that the limited sample size used in these experiments limits our ability to come to a conclusion. Nevertheless, our results suggest that AKT activation downregulates Par-4 function in CaP, which could be a causative factor for CaP progression.

In conclusion, our findings demonstrate that WA efficiently inhibits growth and induces apoptosis in AR-null CRPC cells. WA promotes the nuclear accumulation of FOXO3a, and thus activates its target gene Par-4. These data provide evidence for FOXO3a-dependent transactivation of Par-4 gene activation, which is under tight regulation by AKT. Notably, these results confirm that the AKT/FOXO3a/Par-4 signaling axis can be attractive target for prevention or treatment of CaP.

## Materials and Methods

### Cell lines, antibodies, and reagent

Human prostate carcinoma cell lines, PC-3 and DU-145, were obtained from American Type Culture Collection and cultured (ATCC) and cultured according to the manufacturer instructions. No authentication was done by authors. Withaferin-A obtained from Nucleus Biopharma Inc. (Calbiochem, Billerica, MA, USA) was dissolved in dimethyl sulfoxide (DMSO), and the cells were treated with DMSO at a final concentration of 0.002%. The following antibodies obtained from Cell Signaling Technology (Danvers, MA, USA) were used for the immunoblotting: AKT, pAKT, FOXO3a, pFOXO3a, p27, 14-3-3, cleaved PARP, cleaved caspase-9, BCL2, and BAX. Antibodies for Par-4, pGSK-3*β*, 14-3-3, HA, GAPDH, *β*-actin, and tubulin, and secondary antibodies of anti-mouse, anti-goat, and anti-rabbit were purchased from Santa Cruz Biotechnology (Santa Cruz, CA, USA). Annexin-FITC kit was purchased from BD Biosciences (San Diego, CA, USA). Propidium iodide was purchased from Sigma-Aldrich (St Louis, MO, USA). Alexa Fluor 488, Alexa Fluor 568, and Prolong gold antifade with DAPI mountant were purchased from Invitrogen (Grand Island, NY, USA). Mammalian expression plasmids for CT-FOXO3a, TM-FOXO3a, DBM-FOXO3a, AKT plasmids, and control vectors were obtained from Addgene (Cambridge, MA, USA). Wt-FOXO3a and Par-4 expression plasmids were obtained from Origene (Cambridge, MA, USA).

### Cell proliferation assay

To confirm the viability of PC-3 and DU-145 cells, MTT assay was performed following the manufacturer's protocol.^47^

### Western blot analysis

The CaP cell lines were treated with the earlier mentioned WA concentrations for the different time points. Cell lysates were used for western blot analysis of the above-specified proteins.^48^

### Real-time quantitative PCR

Total RNA was isolated from DU-145, and PC-3 cell lines by Qiagen RNeasy Kit (Valencia, CA, USA), and 1** ***μ*g RNA was used for cDNA synthesis using the Applied Biosystems cDNA synthesis kit and amplified using SYBR Green supermix (Quiagen Inc, CA, USA). Quantitative RT-PCR was performed as previously published.^48^

### Immunofluorescence microscopy analysis

Fluorescence microscopy was performed on cells grown in 6-well plates containing sterile coverslips and these cells were treated with WA. Subsequently incubated with anti-FOXO3a and/or Par-4 antibody (1 : 500 dilutions) or isotype control antibody and then by fluorescent-tag-conjugated secondary antibodies, using the Nikon T1-Eclipse microscope.^49^

### PLA proximal ligation assay

PLA is a latest proteomics approach for detection of proteins, protein–protein interaction, and post-translational modifications (Olink Biosciences, Uppsala, Sweden). Cells were treated and fixed with anti-FOXO3a and anti-Par-4 antibodies together and allowed to generate rolling circle amplification (RCA) by using specific PLA probe sets. The oligonucleotide sequences were used to detect the proteins that are in close proximity and enzymatically joined to create RCA products. Such immobilized RCA products (RCPs) can be visualized by hybridization of fluorescently-labeled detection oligonucleotides to a sequence encoded in the DNA circle. The amplified RCA corresponds to the amount of oligonucleotides hybridized which produced a bright strong signal, distinguishable from the background in a fluorescence microscope.^50, 51^

### Apoptosis assay

Apoptosis assays were performed with the Annexin V-fluorescein isothiocyanate apoptosis kit-I (BD Pharmingen, San Diego, CA, USA) as described previously.^48^

### Transfection

Cells were plated at a density of 3 × 10^5^/well to achieve a confluence of ∼70% after 24 h. Cells were transfected with 1–2 *μ*g of expression vector using Lipofectamine according to the manufacturer's instruction. Cells were incubated at 37 °C, and the medium was replenished with fresh medium in the presence and/or absence of WA for additional 24 h before the cells were harvested.

### Small interfering RNA

For silencing Par-4 and FOXO3a expression, cells were transiently transfected with 25 nM of Par-4-specific siRNA and 50 nM of FOXO3a-specific siRNA or control non-silencing (NS) siRNA (Santa Cruz Biotechnology). Cells were using *siQuest* transfection reagent (Mirus, Madison, WI, USA) following the manufacturer's protocol.^49^

### ELISA-based DNA–Protein interaction assay

Oligonucleotides representing FOXO3a binding site on the Par-4 promoter sequence were synthesized, and the strands were 5′-labeled using the terminal biotin (Integrated DNA Technologies, Coralville, IA, USA). The biotin-labeled sense strand was annealed to its complementary antisense strand and purified over a Sephadex G-50 column (Sigma-Aldrich). The ER alpha site promoter was used as the positive control.^9^ The prepared nuclear extracts were incubated with Par-4 promoter regions, and oligos bearing FOXO3a-binding sites were utilized.

### DNA pull-down assay

DNA fragment with a wild-type or mutant FOXO3a-binding site and with 5-biotin-labeled forward primers was used. The biotinylated probes were mixed with cell lysates transfected with TM-FOXO3a and DBM-FOXO3a, respectively. This was followed by the addition of streptavidin-agarose beads (Invitrogen) with incubation for an additional 1 h. The streptavidin-agarose beads were washed five times with the binding buffer and continued by the addition of SDS-sample buffer. This complex was subjected to immunoblotting with anti-HA antibody.

### Cloning of the Par-4 promoter

The *Par-4* sequence between +1 and −2907 bps was utilized to generate two luciferase reporter plasmids with 5'-UTR +1 to −2900 (2.9 KB) and without 5' UTR −762 to 2900 bp (2.1 KB). Amplification of the Par-4 promoter fragment was done by PCR using primers specific for the region of interest. PCR was conducted using Phusion high fidelity PCR master mix (NEB, MA, USA). Amplified product was digested with restriction enzymes, *Kpn*1 and *Xho*1; and same sites were used to nick the reporter vector to get sticky ends and were utilized for ligation. Ligation mixture was transformed and screened using ampicillin selection. The plasmid DNA fragment encoding the Par-4 region in pGL3-Basic luciferase reporter vector (Promega, Madison, WI, USA) was validated by sequencing.

### Chromatin immunoprecipitation

Ch-IP was performed as described in Cell Signaling ch-IP kit with some modifications. CaP cells over expressing FOXO3a were treated with formaldehyde (1%) for 10 min at 37 °C to cross-link proteins to DNA. Soluble chromatin was subjected to overnight immunoprecipitation with anti-FOXO3a antibody. Following immunoprecipitation and elution, the eluent was heated to reverse the cross-link and DNA was isolated and subjected to amplification. PC-3 cells transfected with/without FOXO3a were also subjected to Ch-IP with the anti-FOXO3a antibody. The PCR products were resolved on a 1.5% agarose gel, stained with ethidium bromide.

### Dual-luciferase reporter assay

To perform the dual-luciferase reporter assay, Par-4-luc promoter constructs and Deleted-Par-4 constructs (1–3 *μ*g) and Renilla luciferase (100*η*g) vectors were cotransfected using Lipofectamine 2000 (Invitrogen). The reporter assay was performed using the Dual-Luciferase Reporter Assay System from Promega.^49^

### Xenograft studies

All animals were housed under pathogen-free conditions, and experiments were performed in accordance with Institutional Animal Care and Use Committee approval, Texas tech university health sciences center, El Paso, Texas. Balb/c athymic nude mice (*nu*/*nu*) were purchased from the Jackson Laboratory (Bar Harbor, ME, USA) and used at 6–8 weeks of age. For tumor xenograft studies, pCMV/DU-145 or AKT/DU-145 cells (2.5 × 10^6^) in a 50-*μ*l final volume matrigel matrix were injected subcutaneously into separate flanks of the mouse (6–8 animals). The mice were monitored twice weekly, and tumor volumes were measured once a week.

### Immunohistochemical analysis

Human Prostate cancer TMA (Cat no. PRC 961) was purchased from Pantomics (Richmond, CA, USA). Each slide has 48 cases from hyperplastic and cancer tissues with progressive Gleason scores and TNM stages in duplicates. For each grade, the TNM classification is provided in the product data sheet. PCa tissue array slide was stained with primary antibody for AKT, pAKT (ser473), Par-4, FOXO3a and pFOXO3a (ser253) followed by secondary antibody incubation, and was analyzed under a light microscope. The TMA slide was viewed and scored by a pathologist.

### Statistical analysis

Data are represented as the mean±standard errors mean (S.E.M.). Significant differences between the groups were determined using the unpaired Student's *t*-test (*P*<0.05). All statistical analyses were performed with GraphPad Prism 6.0a software (GraphPad Software Inc., La Jolla, CA, USA) or Microsoft Excel.

## Abbreviations

FOXO3a: Forkhead box O-3a
WA: Withaferin-A
Par-4: Pro-apoptotic response-4
CRPC: castration-resistant prostate cancer
FasL: Fas ligand
TRAIL: tumor necrosis factor-related apoptosis-inducing ligand
DBM: DNA binding mutant
CHX: cyclohexamide
TMA: tissue microarray
BPH: benign prostatic hyperplasia
KD: kinase dead
p27: Protein27
DBM: DNA binding mutan
pAKT: phosphorylated AKT
DMSO: dimethyl sulfoxide
GSK3*β*: glycogen synthase kinase 3-beta
PARP: poly (ADP-ribose) polymerase
MTT: Tetrazolium dye MTT 3-(4,5 dimethylthiazol-2-yl)-2,5-diphenyltetrazolium bromide
WT: wild type
RCA: rolling circle amplification
UTR: untranslated region
Bim: BH3-only proteins
Bcl-2: B-cell lymphoma 2
