# Supplementary material for: Inhibition of AKT promotes FOXO3a-dependent apoptosis in prostate cancer
Source: Cell Death Dis. 2016 Feb 25;7(2):e2111–. doi: 10.1038/cddis.2015.403 (PMC4849149; doi:10.1038/cddis.2015.403)
Supplement: Supplementary Figure Legends [file cddis2015403x1.pdf]

Supplement Figures

Figure S1. WA inhibits AKT function.

(A) PC-3 and DU-145 cells were treated with WA (2μM) at different time points as indicated earlier and western blot analysis were performed for pAKT(Ser473) and AKT, Par-4 and pGSk3a/b and *β*-Actin or GAPDH used as loading controls.

(B) PC-3 and DU-145 cells were treated with WA for the different time points and RNA isolated were evaluated for Par-4 expression by RT-PCR, with 18S/actin as a control.

(C) PC-3 and DU-145cells were co-transfected with Par-4 promoter reporter construct along with renilla CMV as a transfection control, and the cells were left with or without WA treatment. After 24 hours cells were harvested and assayed for luciferase reporter activity. Bars represents mean of three experiments with SE.

(D) Effect of different concentrations of WA treatment on the viability of PC-3 and DU-145 cells for 24 h as determined by MTT. The cells were treated with DMSO (control) or with the indicated concentration of WA for 24h. Significant difference from control values was indicated at $*P<0.05$, $**P<0.005$ and $***P<0.0001$ (students T-test).

Figure S2. Functional mutant FOXO3a fails to activate Par-4 function

(A) Cells were transiently transfected with CT-FOXO3a and empty vector. After transfection, cells were treated with or without WA and total cellular lysates were extracted and subjected to western blot analysis fort pAKT, AKT, FOXO3a, pFOXO3a and Par-4 proteins. *β*-Actin was used as a loading control.

(B) FOXO3a and Par-4 proteins in WA-treated or control cells were immunostained with primary and the corresponding FITC- or TRITC-conjugated secondary antibodies followed by detection using confocal microscopy. Green signals indicate FOXO3a, whereas red signals indicate Par-4. Nuclei were counterstained with DAPI. Representative images of each sample are shown.

(C) PC-3 cells were co-transfected with Par-4 promoter luciferase reporter construct, CT-FOXO3a expression plasmid construct with renilla CMV as transfection control, and /or treated with WA. After 24h cells were harvested and assayed for luciferase reporter activity.

(D) The cells were treated with DMSO (control) or the indicated concentration of WA for 24h. Bars represents mean of three experiments with SE Significant difference from control values was indicated at $P<0.05$ (students T-test).
