# Supplementary figures and images for: Inhibition of AKT promotes FOXO3a-dependent apoptosis in prostate cancer
Source: Cell Death Dis. 2016 Feb 25;7(2):e2111–. doi: 10.1038/cddis.2015.403 (PMC4849149; doi:10.1038/cddis.2015.403)

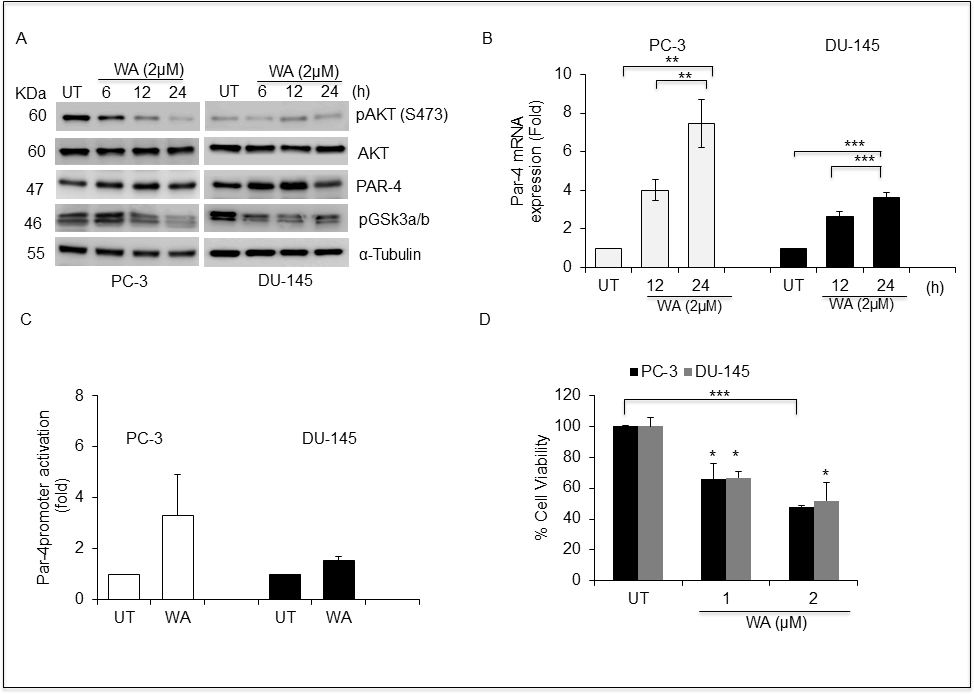

Supplement: Supplementary Figure 1 [file cddis2015403x2.tif]

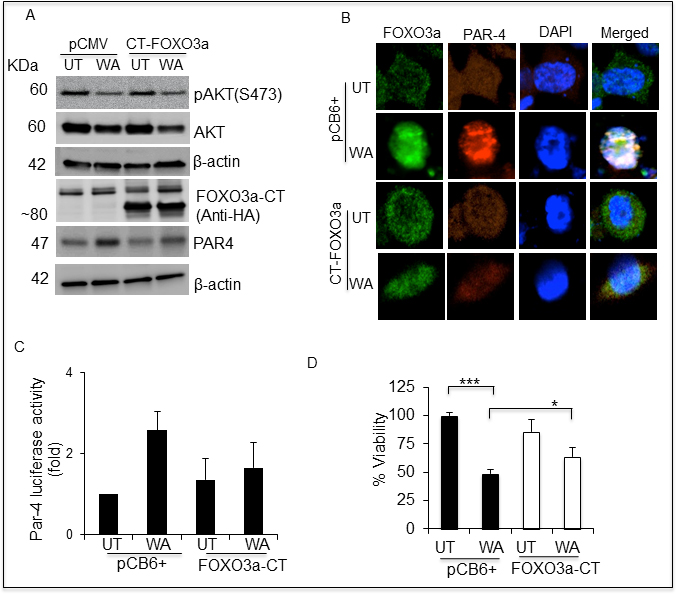

Supplement: Supplementary Figure 2 [file cddis2015403x3.tif]
